# Large closed-basin lakes sustainably supplied phosphate during the origins of life

**DOI:** 10.1126/sciadv.adq0027

**Published:** 2025-02-19

**Authors:** Craig R. Walton, Jihua Hao, Maria Schönbächler, Oliver Shorttle

**Affiliations:** ^1^ Institute of Astronomy, University of Cambridge, Madingley Road, Cambridge CB3OHA, UK.; ^2^ Department of Earth Sciences, Institute für Geochemie und Petrologie, ETH Zurich, NW D 81.2, Clausiusstrasse 25, Zurich 8092, Switzerland.; ^3^ Deep Space Exploration Lab/CAS Key Laboratory of CrustMantle Materials and Environments, University of Science and Technology of China, 96 Jinzhai Rd., Hefei 230026, China.; ^4^ State Key Laboratory of Lithospheric and Environmental Coevolution, University of Science and Technology of China, 96 Jinzhai Rd., Hefei 230026, China.; ^5^ Department of Earth Sciences, University of Cambridge, Downing Street, Cambridge CB23EQ, UK.

## Abstract

The origin of life on Earth required a supply of phosphorus (P) for the synthesis of universal biomolecules. Closed lakes may have accumulated high P concentrations on early Earth. However, it is not clear whether prebiotic P uptake in such settings would then have been sustainable. We show that large closed-basin lakes can combine high P concentrations at steady state with extremely high rates of biological productivity. Our case study is Mono Lake in California, which has close to 1 millimolar dissolved P at steady state despite extremely high rates of biological productivity, in contrast to smaller closed basins where life is scarce. Hence, large closed-basin lakes offer an environment where high rates of prebiotic P productivity can plausibly coexist with high steady-state P concentrations. Such lakes should have readily formed on the heavily cratered and volcanically active surface of early Earth.

## Teaser

Large closed-basin lakes offer a sustainable source of phosphate that may have met the strict demands of prebiotic chemistry.

## INTRODUCTION

Phosphorus (P) is a critical element for all known forms of biochemistry, playing vital roles in metabolism, cell structure, and information-encoding polymers, e.g., DNA (*1*). However, the environmental conditions that rendered P sufficiently available in aqueous solution to promote the chemical origins of life remain uncertain, i.e., the P problem (*2*). Evidence from experimental prebiotic chemistry indicates that high (100 mM) concentrations of dissolved inorganic P (*3*–*8*), which rarely occur in natural environments, are needed to synthesize biomolecules, wherein P is used up directly in reactions and also provides a general acid base catalyst and a pH buffer for prebiotic chemistry. This contrast between the general scarcity of dissolved P in nature and the apparently high P demand of prebiotic chemistry is often referred to as the so-called prebiotic P problem (*2*, *9*). However, a high initial concentration of P is not necessarily enough for the origin of life: We must also be able to sustain the emerging prebiotic chemistry.

Sustaining prebiotic chemistry means that the system somehow avoids using up its own substrate and therefore becoming self-limiting. Even before the emergence of the first cells, the high P concentrations needed for many reactions to occur must have been sustained despite uptake by prebiotic reactions. This is challenging, as most environments on Earth today have low P concentrations due to a combination of limited P supply and draw-down by life. As a result, life in most environments on Earth today is P limited (*10*). Prebiotic analogs of these common environments would have therefore likely run into P limitation as demand grew, becoming self-inhibiting. Therefore, we can instead consider some relatively rare environments where P is not limiting, i.e., with a sustainable P supply.

On Earth today, phosphate fluxes (P per km^2^ year^−1^) are crucial in determining total biological productivity [carbon (C) per km^2^ year^−1^] (*10*). The same will hold true for prebiotic productivity—defined here as the flux of dissolved P incorporated by prebiotic chemistry per unit area or volume per unit time, e.g., P per km^2^ year^−1^ or P per liter year^−1^—with the caveat that many prebiotic reactions may not be able to efficiently harness dilute P sources. In this way, environments with low P fluxes will not be able to sustain high rates of prebiotic productivity. Given the short lifetimes of many prebiotically relevant species to hydrolysis (*11*) and the likelihood of losing P during reactions to inert side products, this may be a severe problem for defeating the arithmetic demon in prebiotic chemistry (*12*).

Recently, closed-basin lakes have been identified as an environment that, if present on prebiotic Earth, may plausibly have accumulated large concentrations of dissolved P, i.e., a stockpiling route out of the P problem (*13*). In closed lakes, P sources to an aqueous reservoir via weathering are balanced by inefficient internal sinks and a lack of basin outflow (*13*). Hydrological balance may be maintained by evaporation, or the basin may repeatedly evaporate entirely. As a result, high concentrations of P may be obtained. This scenario plays out today in soda lakes: closed-basin lakes in which P is highly soluble (*13*). However, the emergence of prebiotic chemistry—and ultimately life—itself becomes an internal sink for P in the basin.

The question then arises: Are closed-basin lakes capable of both initiating and sustaining prebiotic chemistry? In this context, the generally limited rate of P input to closed-basin lakes may be concerning, as this will be a primary factor determining the steady-state concentration achieved as internal basin sinks become increasingly efficient as well as the productivity of the system. In other words, we must identify settings that can both build up high concentrations of P, to enable prebiotic chemistry to run, and can sustain these high concentrations when the chemistry becomes productive, and that can actually support high levels of productivity at all.

Here, we examine the conceptual interplay of P sources and sinks pertinent to prebiotic closed lake scenarios. We estimate P availability and sustainability in prebiotic closed basin environments by combining empirical observations of modern systems with a steady-state model of P concentrations as a function of prebiotic uptake efficiency, combined with corrections for the absence of biological activity. We show that the high rates of prebiotic productivity alongside high steady-state P concentrations could have been achieved in high inflow closed lakes on early Earth.

## RESULTS

### Sources of P

**Fig. 1.**
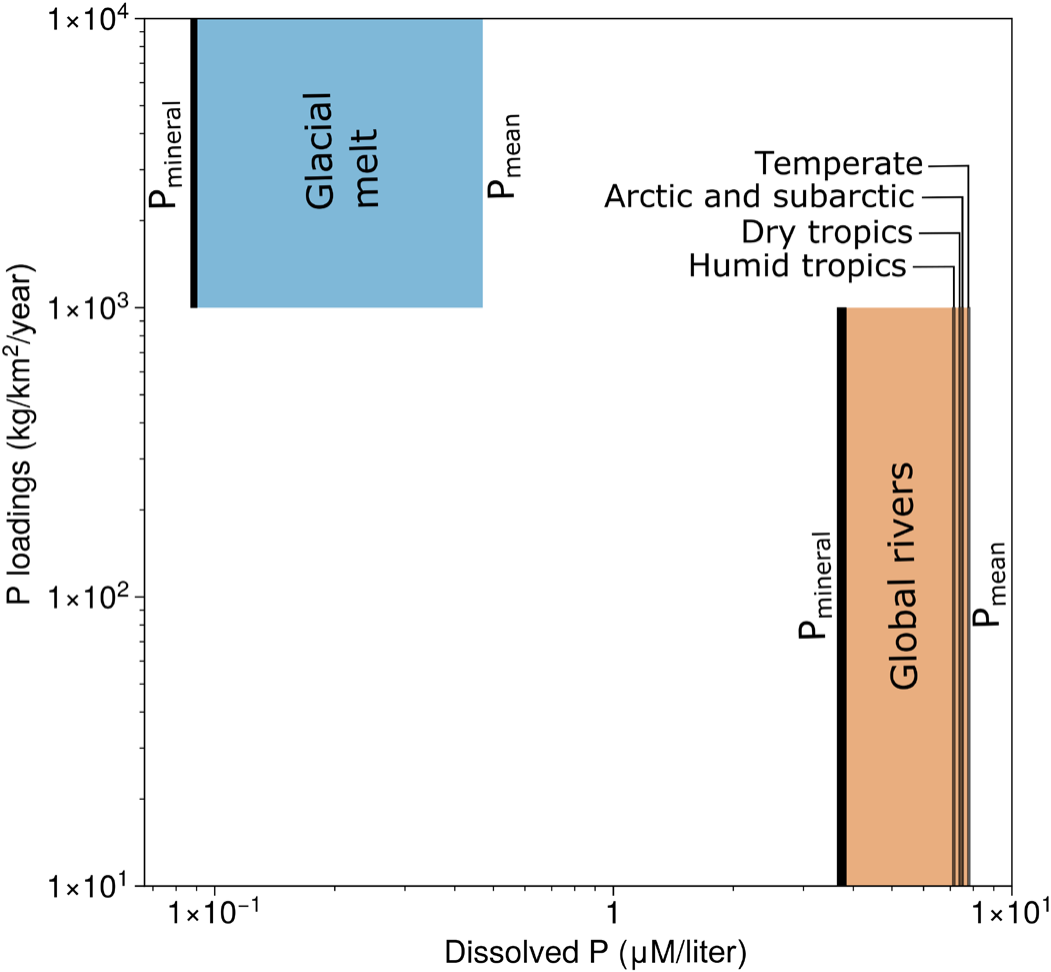
Dissolved P concentrations and fluxes in modern weathering systems. Glacial data are from (*16*); riverine data are from (*18*). P_mineral_ refers to dissolved P, and P_mean_ is the average total P. On the modern Earth, high P fluxes do not produce high dissolved P concentrations. Starting prebiotic chemistry requires the latter, but sustaining it requires the former.

P surface weathering fluxes vary by more than two orders of magnitude across Earth’s surface today, driven by variations in local crustal P content, lithology, and erosion rate (Fig. 1) (*14*, *15*). Proglacial environments yield P weathering fluxes several orders of magnitude higher than in most rivers (Fig. 1) and also sustain high rates of primary productivity (*16*). However, these large variations in P fluxes do not translate into equal variation in the P concentrations of runoff waters. High weathering rates strongly correlate with runoff volumes, resulting in dilution of high P fluxes (Fig. 1). P concentrations in glacial runoff are actually lower than it is in average rivers of any catchment type (Fig. 1). This result lies in stark contrast to the fact that glacial runoff creates among the largest known P fluxes on Earth (Fig. 1).

Another complication for evaluating P supply to closed basins is the complex activity of Earth’s present-day biosphere, which of course contaminates all such extant examples of these unusual environments. In particular, animal species can act as vectors to input vast quantities of nutrients into specific environments. In the case of soda lakes, migratory birds often inject large quantities of P each year—in some cases, representing almost all of the P input (within error of 100%) (*17*). We adopt a correction for the observed partitioning of P sources when considering specific lakes (see the next section), allowing us to properly evaluate the P fertility of prebiotic analogs of modern soda lakes (*13*). Similarly, biology acts to break down and recycle P from sinking organic matter, promoting higher P availability than would otherwise occur for a given rate of biological productivity (*10*). However, as we will now demonstrate, it is basin hydrology and geometry that play the most decisive roles in determining the availability of P in closed basins.

### Sinks of P

**Fig. 2.**
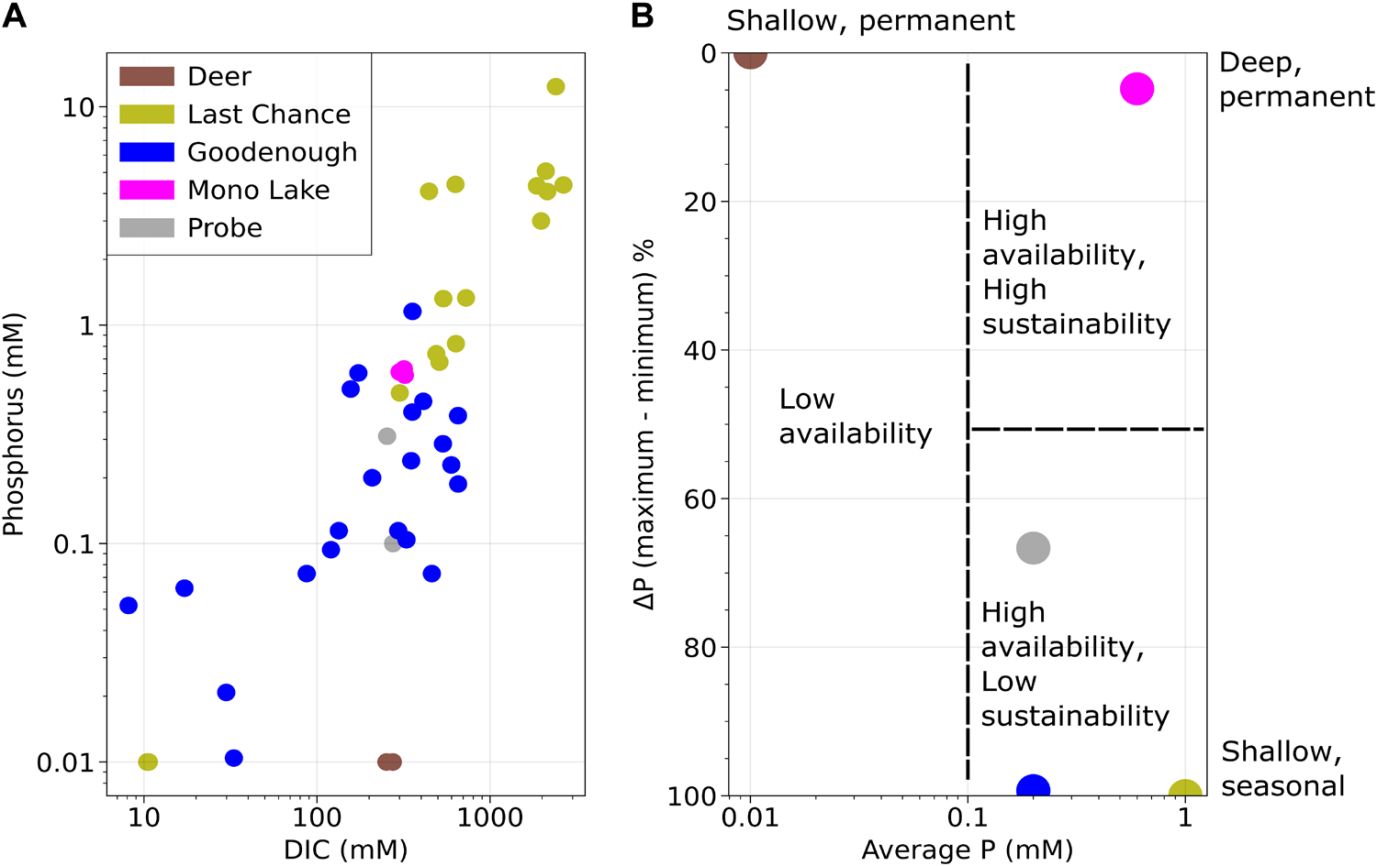
P availability in soda lakes. (**A**) Dissolved P concentrations in a series of soda lakes. Measurements are from a variety of studies and were made at different points in time (*19*, *20*). Data for Last Chance and Goodenough lakes span a full wet to dry season cycle. (**B**) Annual variability of P concentration in soda lakes as assessed by percentage change between maximum and minimum reported concentrations. Classes of soda lake with otherwise similar P concentrations can be discriminated between on the basis of these often extreme seasonal changes.

**Fig. 3.**
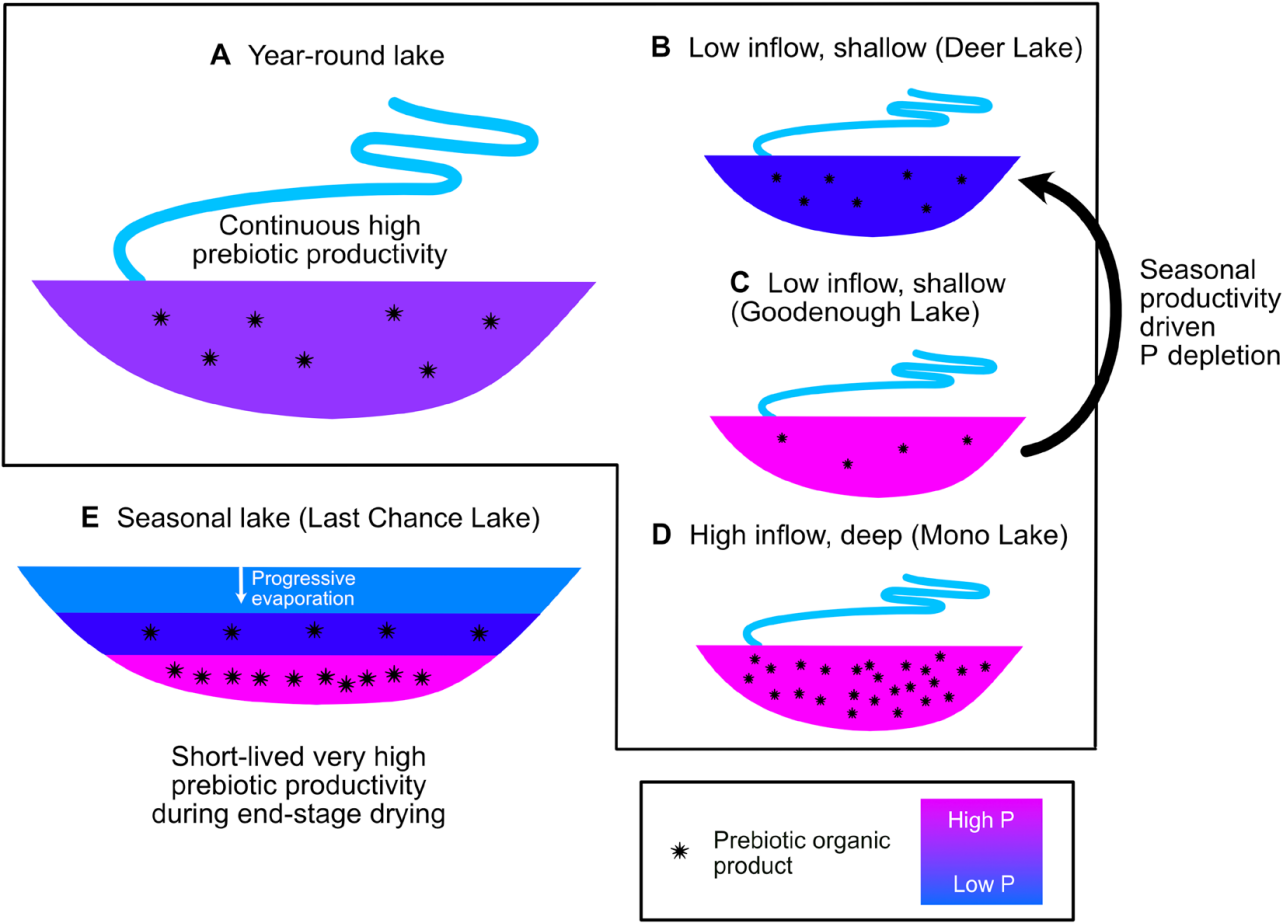
Hydrology plays a large part in determining P availability and sustainability. (**A**) Large and deep closed lakes, sustained by continuous inflow, ensure that P concentrations are sustained even with efficient sinks, e.g., life or extensive prebiotic chemistry. Shallow permanent lakes can have low P availability and relatively high productivity if internal P sinks are large compared to sources (**B**), or high P availability and relatively low productivity if P sinks are low compared to sources (**C**). Sustainability of steady-state dissolved P in these lakes is low, as demonstrated by the example of Goodenough Lake (*19*), which only has high P concentrations when biological sinks are scarce and shows huge seasonal swings in dissolved P content. (**D**) Extremely high productivity alongside high P concentrations may be achieved in the largest end-member lakes, e.g., Mono Lake, California. (**E**) Seasonal lakes transiently concentrate P to the levels required for prebiotic chemistry during evaporation. However, the basin will then dry out completely. This may be considered fatal or advantageous for prebiotic chemistry depending on the specific synthesis pathway being considered.

First, considering empirical observations of the P concentrations of soda lakes (Fig. 2), we can define several different classes of basin (Fig. 3). First, there are lakes that display immense variation in measured P concentration (e.g., Goodenough, Last Chance; Fig. 2). These multiple order of magnitude variations occur during annual desiccation and rehydration cycles, which massively concentrate dissolved inorganic carbon and P during the evaporative phase coupled to a lack of primary production driven P uptake (Last Chance) or during seasonal booms in primary productivity, which may conversely deplete dissolved P (Goodenough). In the case of Goodenough Lake, the minimum P concentrations observed can be very low indeed, falling below detection by several analytical methods and being indistinguishable from the P content of inflowing runoff (Fig. 2) (*18*).

Seasonal lakes face some challenges for hosting high rates of prebiotic productivity at steady state. (i) Fully seasonal lakes face an inherent interruption of productivity during the dry phase (Fig. 3E). That is not to say that the high P concentrations attained up to the point of desiccation would not have reliably driven prebiotic chemistry (*13*, *19*), and it is also true that some of the most P-rich seasonal lakes never fully evaporate, e.g., Last Chance lake (*13*). Nonetheless, (ii) the P uptake driven by that prebiotic chemistry would have been potentially self-limiting if P uptake efficiency by prebiotic chemistry was high compared to the low P input rate for such closed basins. This may be implied by the fact that small soda lakes with higher rates of biological productivity have much lower P concentrations than their unproductive counterparts (*20*). Last, (iii) molecules produced during the high P dry season would be subject to long periods of relative dilution during the low P wet season and hence be exposed to destructive hydrolysis (Fig. 2) (*21*). In this way, seasonal soda lakes present a compelling scenario for intermittently productive prebiotic chemistry that may in some cases be coupled to wet-dry cycles but may be less well suited to hosting high prebiotic productivity at steady state. Here, we consider whether large closed-basin lakes may have more sustainably supplied phosphate during the origins of life.

We define three classes of permanent soda lake. The first class is present throughout the year yet is shallow and has minimal riverine input—and therefore limited P input. Some of these lakes are in turn strongly P limited, e.g., Deer Lake (Fig. 3, A and B). Deer Lake would satisfy the requirement for continuous prebiotic chemistry from the perspective of having a constant input of P. However, this input is very small. Either P would never be concentrated enough to initiate prebiotic chemistry in a Deer Lake scenario or, if abiotic sinks for P were essentially nonexistent, prebiotic P concentrations would gradually build up to the threshold needed to initiate reactions but the rate of productivity achieved would be extremely limited (see Discussion).

The second class of permanent soda lake has low inflow, is shallow, and is strongly seasonal in terms of productivity, e.g., Goodenough Lake (Fig. 3C). Goodenough Lake varies in P concentration from as high as 1 mM to orders of magnitude lower than this (Fig. 2), apparently due to the seasonality of biological productivity in the lake. In other words, the small reservoir size of this type of lake makes it vulnerable to P depletion when productivity is initiated (*20*).

Last, we highlight a third class of high inflow and deep closed-basin soda lakes, e.g., Mono Lake (Fig. 3D). These lakes exhibit P concentrations that are high (just below 1 mM) throughout the year (Fig. 2), equal from surface to depth (well mixed), and also—critically—support immense levels of modern biological productivity, e.g., 100 mM C per m^2^ per day (*18*, *22*). It is this class of closed basin that might in principle be most capable of sustaining high rates of prebiotic productivity, owing to a favorable intersection of large and continuous P inputs, a large reservoir size, and efficient evaporation.

### Prebiotic P availability in closed basins

We now consider P availability in prebiotic analogs of each of these soda lake end-members (Figs. 2 and 3). Using modern examples to inform us about the prebiotic case is complicated by two factors: nutrient recycling and non-riverine nutrient vectors. Today, oxidative remineralization of organic matter results in extensive P regeneration (*23*) and avian nutrient vectors supply more than 50% of total P in many soda lakes (*17*). These processes sustain higher P concentrations in soda lakes than would otherwise abiotically occur given the same environmental conditions.

We construct a simple steady state model for P concentration that considers weathering input as well (pre-)biotic sinks for P (see Materials and Methods). We inform our model with data from a number of modern soda lakes, considering annual inflow, lake volume, total annual P input, internal P recycling efficiency, external P vectors, P turnover time, and mean dissolved P concentration in the basin.

**Fig. 4.**
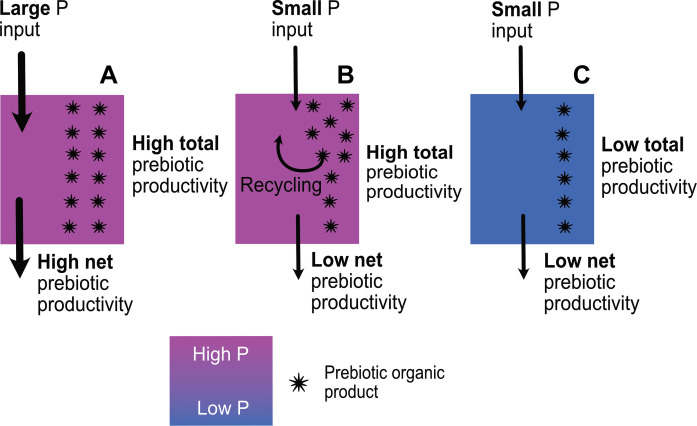
Scenarios for P-limited prebiotic productivity at steady state. Each scenario assumes constant P uptake efficiency. (**A**) A basin with a large P input will have high net productivity. However, total productivity may nonetheless be relatively low if internal recycling efficiency is low. (**B**) Small P inputs will yield low net productivity. Total productivity and steady-state P concentrations might nonetheless be high if internal recycling is efficient, as shown. (**C**) Small P inputs coupled to limited recycling will support low total and net productivity as well as low steady-state P concentrations.

Before proceeding, it is helpful to review how these factors interact to determine the productivity, steady-state P availability, and efficiency of P uptake, described here in terms of the P turnover rate, in closed-basin lakes. We can characterize the production rate of prebiotic/biomolecules in a basin in terms of total and net productivity (Fig. 4). Net productivity (millimole P liter^−1^ year^−1^) describes the mass of, in this case, P in organics produced each year that are exported permanently and not recycled. Total productivity (millimole P liter^−1^ year^−1^) in turn simply describes the mass of organics produced each year, i.e., including any molecules that are ultimately destroyed. The turnover rate of P in the basin (units of per year) is then calculated by dividing the net productivity by the concentration of P in the basin at steady state.

Total productivity is set by the combination of P input and internal P recycling by life. At steady state, net productivity must always be equal to the total P input (Fig. 4). Hence, a small basin with extremely efficient recycling, i.e., low turnover times, could well have higher total productivity than a large basin with little to no recycling (Fig. 4, B and C), but net productivity will always be higher in whatever basin has the higher P input (Fig. 4A). The requirement for mass balance between sources and sinks of P at steady state means that, in a system with minimal abiotic sinks, the P concentration will adjust according to the input flux and the efficiency of internal recycling (Fig. 4).

**Fig. 5.**
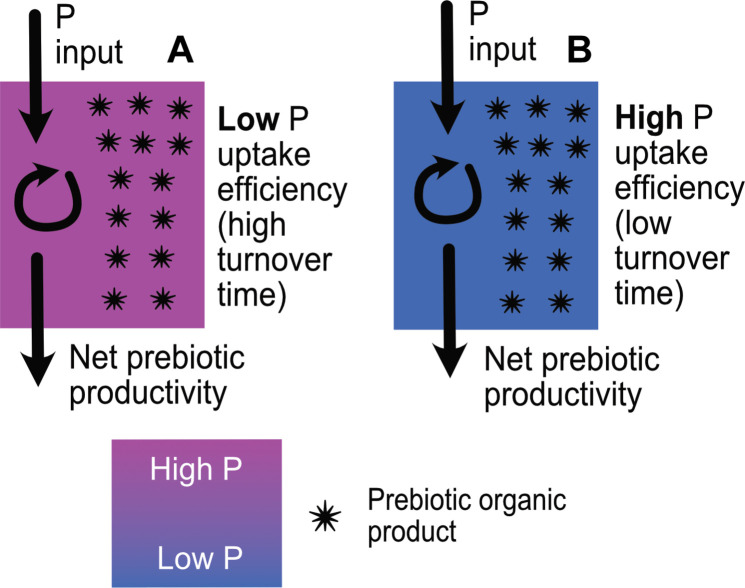
Dependency of steady-state P concentration on uptake efficiency. (**A**) Low P uptake efficiency leads to high steady-state P concentrations. (**B**) High P uptake efficiency leads to low steady-state P concentrations if recycling efficiency and P input are kept constant. Both total and net prebiotic productivity are equal in the two scenarios shown.

In contrast, consider two basins with identical P input but differing efficiencies of P uptake (Fig. 5, A and B). The basin in which P uptake is most efficient will have lower steady-state P concentrations (Fig. 5B), yet total and net productivity will be identical in each basin. Nature is of course somewhat more complicated than this. However, these rules provide a useful starting point to understand the pros and cons of each class of soda lake for hosting P-dependent prebiotic chemistry.

**Fig. 6.**
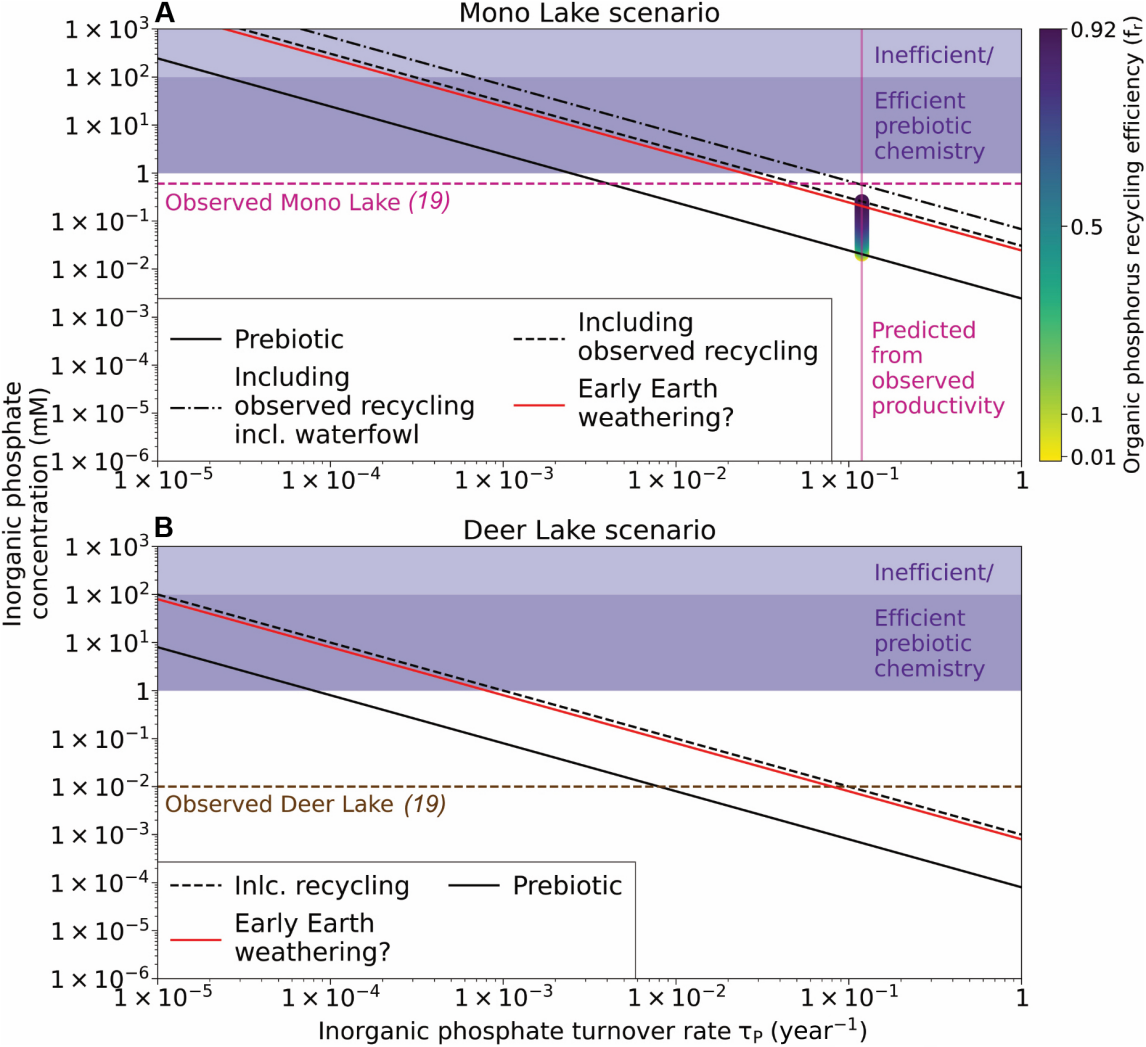
P availability in extant and prebiotic analogs of soda lakes. (**A**) Dissolved P steady-state concentrations for a closed-basin lake with the hydrological and geochemical characteristics of Mono Lake, as a function of P turnover rate (τ_P_). An estimate of the present-day turnover rate is indicated. Estimated steady-state P concentration as a function of turnover rate are shown, as are separate estimates after correcting for the prebiotic absence of nutrient vectors and biological recycling, as well as the possibility of enhanced weathering on early Earth. The average present-day steady-state P concentration in Mono Lake is indicated. (**B**) Dissolved P steady-state concentrations for a closed-basin lake with the hydrological and geochemical characteristics of the P-poor Deer Lake. In each case, the parameter space needed for prebiotic chemistry is indicated by the shaded labeled region. The average present-day steady-state P concentration in Deer Lake is indicated.

Figure 6 explores the parameter space for steady-state P concentrations in soda lakes as a function of the turnover rate of P in the system: the fraction of P that leaves the basin in a specified interval. Turnover rate tells us about the efficiency of P sinks. If at steady state, a tiny fraction of P leaves the lake per unit time, then P sinks are necessarily inefficient, and vice versa. The observed P concentrations of the high and low input soda lakes from Fig. 2—Mono Lake and Deer Lake—are indicated. Estimates of steady-state P concentration in each case are given as a function of P turnover rate. In this way, we can correct for the present-day high efficiency of P uptake by biology to explore P concentrations in a putative low efficiency prebiotic regime, as well as explore how P recycling efficiency likely affect P concentrations in each lake today.

In Fig. 6A, the observed productivity and P concentration of the high inflow soda basin Mono Lake in California is used to estimate turnover rate for P in the basin. A relatively high value is obtained. First, correcting to remove the effect of animal nutrient vectors as well as modern-day efficient P recycling, which operates because of biological breakdown of sinking organic matter via oxidant-powered respiration pathways (*24*), sees steady-state P concentrations drop from around 1 to 0.01 mM. However, we can consider that prebiotic chemistry is likely to be less efficient than biology at taking up P. The turnover rate would be correspondingly low, and steady-state P concentrations should be fixed at whatever high concentration represents the kinetic threshold for the reaction to take place at all.

Experimental results show that phosphate is participant in prebiotic chemistry when using concentrations of around 100 mM (*3*, *4*, *7*). The lower concentration thresholds are poorly explored, but we cannot at present say with any confidence that concentrations below 100 mM are relevant, at least when considering orthophosphate. While P inputs to a prebiotic lake analog may be lower than today—given the same environmental conditions due to the absence of avian nutrient vectors and that recycling efficiency might also be much lower—inefficient P uptake (in the absence of effective abiotic sinks) could nonetheless result in sufficient P concentrations to sustain continuous prebiotic chemistry (Fig. 6A).

In principle, similar outcomes hold true for low P-input soda lakes such as Deer Lake. While data availability for such lakes are currently limited, we can place rough bounds on present-day productivity on the basis of what is known about the steady-state P concentrations, inflow volume, and lake volume (Fig. 6C). It appears that the turnover rate of P in Deer Lake may be similar to Mono Lake despite their major differences in steady-state P concentration. Yet, we can also see that Deer Lake is in many ways not as competitive as Mono Lake in a prebiotic context.

**Fig. 7.**
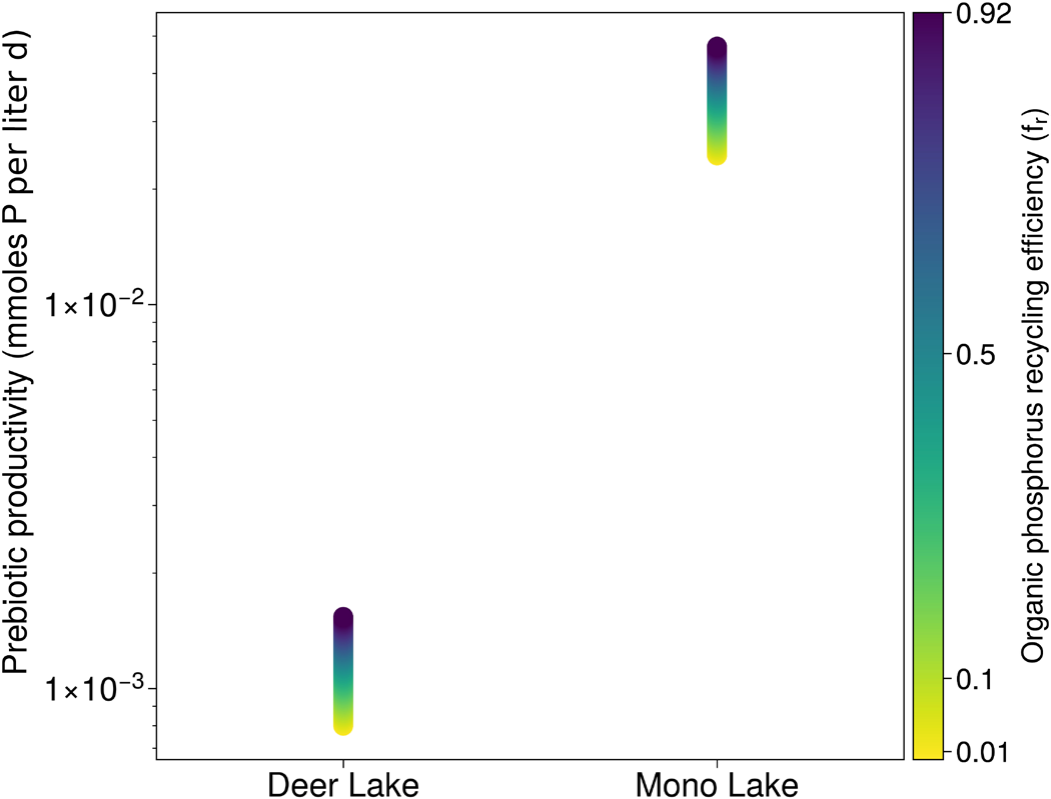
Major differences in the potential prebiotic productivity of soda lakes. Potential prebiotic productivity is the burial rate of P in the system, assuming that prebiotic chemistry is the sole sink of P in the system and accounting for the possibility of internal P recycling. The maximum rate of synthesis of organic P in a Deer Lake scenario is offset from the maximum rate in a Mono lake scenario by more than one order of magnitude. We highlight a range of P recycling efficiencies from near zero to the approximate efficiency of recycling identified in Mono lake today. Note that this calculation assumes a steady-state scenario for Deer Lake.

For example, consider the turnover rate needed to obtain the same steady-state P concentration in each basin (Fig. 6). This rate must be several orders of magnitude lower for Deer Lake than Mono Lake to achieve parity in dissolved steady-state P concentrations, i.e., a much less efficient uptake of P by organic sinks. Hence, P is much more readily available to prebiotic chemistry in Mono Lake. Even minor abiotic P sinks are in the case of a Deer Lake analog likely to compete with organic P sinks, limiting the steady-state P concentrations (*25*, *26*). Less evaporated and lower alkalinity lakes such as this will have a lower ceiling for dissolved P than Mono Lake (*13*). Even if this were not the case, the total productivity in a Mono Lake analog would outstrip that in a Deer Lake analog by more than an order of magnitude for any given P recycling efficiency (Fig. 7).

For both lakes, even if the kinetic threshold for prebiotic P productivity is much higher than the concentrations observed in Mono Lake or Deer lake today—say, 100 mM—it could be achieved at steady state in such a basin with continuous inflow provided that abiotic sinks are minimal. However, it is clear that such inefficient abiotic sinks would require surface conditions on Earth and thus soda lake geochemistry that strongly differs to those found today, since even the most highly evaporated and poorly productive lakes, e.g., Last Chance Lake, do not achieve 100 mM dissolved P (Fig. 2) (*13*).

### Prebiotic P recycling pathways

Given present-day P inputs and turnover rate, P concentrations in Mono Lake would be expected to be around 0.025 mM—more than one order of magnitude lower than observed today (Fig. 6A). The difference between model and observation is explained by modern efficient recycling of P—up to 92% (*27*)—as well as by the input of large quantities of P by more than 2 million migratory birds each year (*28*), which we estimate by comparing the shortfall of our steady state model versus observed P concentrations (Fig. 6A). While 0.025 mM P—despite high productivity—is nonnegligible in a prebiotic context (*21*), it is substantially lower than apparent threshold concentrations of phosphate required for prebiotic chemistry of 100 mM (*4*). In contrast, a Deer Lake analog scenario under the same conditions of prebiotic P uptake would have less than 1 μM P at steady state.

Our calculations show that an approach to modern equivalent P turnover rates may require the near shutdown of any reactions requiring high steady-state P concentrations in both Mono Lake and Deer Lake analog scenarios. However, we can consider whether the conditions of early Earth helped to offset the absence of biological nutrient recycling and avian nutrient vectors, fostering more favorable conditions for prebiotic chemistry in prebiotic closed basins. We consider two possible and nonmutually exclusive mechanisms to enhance P availability: enhanced P weathering supply and abiotic P recycling.

In exploring the upper limits on plausible P availability in prebiotic closed basins, we can couple the highest runoff P concentrations from Fig. 1 to the favorable hydrology and geometry (high inflow rate and high surface area) of Mono Lake. We can additionally consider the possibility of high early volcanic outgassing rates, which may have produced higher rates of chemical weathering independent of runoff volumes, i.e., increased P concentration in the streams feeding the lake (*15*, *26*). Together, these effects could amplify P concentrations in a prebiotic Mono Lake twin by around one order of magnitude, yielding concentrations of 0.25 mM (Fig. 6B). This is close to, but still below, the rough lower bound of relevant P concentrations for known early stage prebiotic reaction networks.

**Fig. 8.**
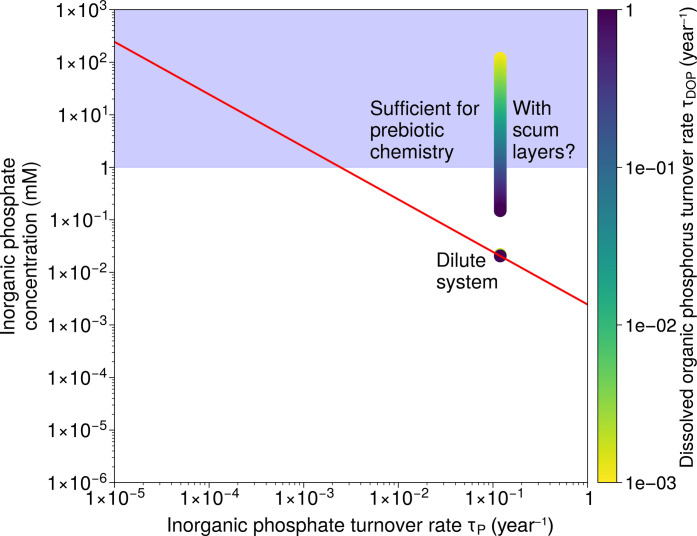
Capacity for photolytic recycling of P in a Mono Lake basin. Even extremely sluggish settling rates of synthesized organic matter do not allow for relevant efficiencies of photolytic P recycling capacity. Shown for comparison is a stratified lake scenario where buoyant multi-compartmentalized “scum” organic layers that allow highly concentrated solutions to be exposed to irradiation. Here, a speculative organic concentration factor of 10^5^ relative to a well-mixed scenario is considered (*33*).

Next, we apply experimental constraints on ultraviolet-induced organophosphorus recycling (*29*). Again, fitting to Mono Lake basin parameters, we vary organic P uptake efficiency but this time account for photolytic P recycling of the resulting dissolved organic P (DOP) (see Materials and Methods). We find that photolytic P recycling cannot provide a relevant internal source of P unless the turnover rate of P-bearing organic matter is extremely sluggish (Fig. 8). While the evidence from soda lakes is that these systems are generally well mixed and turbid (*22*), such that DOP monomers or small polymers may accumulate in solution to high concentrations, our calculations call into question whether this would be sufficient to affect P availability to any great extent, e.g., at the one order of magnitude level. An arbitrary organic concentration factor in surface waters of 10^5^ coupled to dissolve organic P turnover rates of 10^*−*3^ are needed to obtain P recycling fluxes at this level.

## DISCUSSION

### How much P is enough?

Most environments on Earth today are P limited with respect to biological productivity (*10*). Mono Lake is one of the very rare exceptions. Our results show that a Mono Lake analog scenario, i.e., a high-inflow closed soda lake, may present a simultaneous solution to both the concentration and sustainability P problems in prebiotic chemistry, by offering moderately high P concentrations at steady state despite efficient P uptake during prebiotic productivity.

Steady-state P concentrations would remain at around 1 mM (Fig. 6) even if prebiotic P turnover rates were equivalent to those observed in Mono Lake today. If P turnover rates were slower, as seems plausible for prebiotic chemical systems compared to biological ones, steady-state P concentrations could have been as high as 100 mM (Fig. 6). This would be high enough in such a setting to buffer, catalyze, and/or directly participate as a reactant in the initial stages of the chemical network, according to published experimental constraints, although such experiments did not systematically explore the lower limits of P needed for these reactions to progress (*4*, *5*).

Whether or not this concentration could actually have been reached in such a system depends on how soluble P is in the basin, i.e., the onset of abiotic sinks, which is largely determined by how concentrated in carbonate and depleted in divalent cations the lake is (*13*). Considering the spread in P concentrations for a given carbonate and divalent cation composition of a lake (*13*), the argument can reasonably be made that Mono Lake is not currently at P saturation and could accumulate more P than it does today. Even assuming the worst case scenario that Mono Lake sits already almost exactly at phosphate saturation, more carbonate-rich conditions (enabling high phosphate solubility) for a given lake would be induced by higher atmospheric carbon dioxide on early Earth (*13*). We could also consider lakes that are simply slightly more evaporated than Mono Lake is, which would occur if all hydrological factors were kept the same other than a slightly lower local relative humidity. Both options for increasing the dissolved phosphate capacity of closed basins are feasible for early Earth.

Prebiotic reaction schemes do currently use in excess of 100 mM P as standard (*4*). However, there are two points to be made regarding this. The first is an empirical argument. Less than 100 mM may be all that is needed for prebiotic phosphorylation on the basis that these are so far the upper limit for even the most P-enriched environments found on Earth—after a reasonably exhaustive search (*13*). It may therefore be that the “right” reactions and conditions of prebiotic chemistry have yet to be identified, i.e., that we can at least further tune our scenarios to make do with somewhat less available P.

The second argument is that systems like Mono Lake have 1 mM in the entire lake—top to bottom and all year-round, which could have been coupled to processes located anywhere in the basin to further enhance reactions involving P. It is now known that microenvironments can make possible reactions that would not happen in the bulk environment. For example, phosphorylation reactions are enhanced greatly by heated gas bubbles (*6*), which occur in the hydrothermal systems that vent into many permanent soda lakes (*30*). Hence, a 1 mM P lake is a competitive starting point with which to couple chemistry happening in microenvironments. This sort of coupling scenario is essentially impossible for seasonal lakes, since the highest concentrations only occur right at the end of evaporation and cannot therefore intersect a continuous inflow of hydrothermal waters, and more challenging for any other body of water that is relatively dilute in P, e.g., low-inflow lakes, oceans, and so on.

However, while there are a few areas where seasonal lakes have challenges to overcome in terms of supporting prebiotic chemistry, seasonal lakes must contribute to a wider tapestry of linked environments that perform prebiotic synthesis. If the environmental ensemble can be considered stable, then sustainability may be achieved. For example, seasonal lakes adjacent to larger stable lakes may be regularly replenished with solutions rich in P, i.e., restricted basins that operate effectively as a microenvironment of a sustainably P-rich system like Mono Lake. Such seasonal lakes are known to exist adjacent to Mono Lake although have not yet attracted detailed geochemical study. In this way, the advantages of large stable lakes with sustainable P supply and small seasonal lakes with higher peak P concentrations may be linked.

### Supply and demand at the origins of life

Achieving productive and sustainable chemistry in a Mono Lake scenario as described above requires enhanced P sources relative to today and high abiotic recycling efficiencies (Fig. 6)—the latter of which does not immediately appear to be plausible (Fig. 8). However, as reiterated at various points here, prebiotic chemistry may well have been far less efficient than biology at taking up available P. Hence, even if we assume no prebiotic recycling of P, a Mono Lake scenario can still support 1 mM steady-state dissolved P if turnover rates are simply 10 times lower than they are in Mono Lake today (Fig. 6). Smaller lake (Deer Lake and Last Chance Lake) scenarios can also readily accumulate high P concentrations but require slower P turnover rates (Fig. 6) and cannot support equally high prebiotic productivity (Fig. 7). For context, assuming that productivity could be totally delayed until lake levels are at their lowest in the dry season and P concentrations are at their peak, the levels of biological productivity found today in Mono Lake (*18*) would fully deplete a Deer Lake system in P in around 1 to 10 days and would fully deplete a Last Chance Lake system (the highest reported dissolved P concentrations on Earth) in under 1 day.

There is however the caveat that prebiotic productivity will not necessarily operate in a directly analogous manner to biological productivity. For example, the latter is mainly confined in the lake photic zone. This renders the dissolved P inventory of a large reservoir such as Mono Lake hard to perturb in contrast to that in a shallow lake, e.g., Goodenough (Fig. 2). If prebiotic production takes place throughout the entire lake system rather than just the photic zone, the empirical argument for a Mono Lake environment being harder to deplete in P than a Goodenough lake scenario becomes weaker. Qualitatively, we would argue that large volume lakes are inherently less likely to be P-limited than small ones. Depleting a lake in P via prebiotic chemistry requires a sufficiently large source of carbon (C), for example, such that prebiotic productivity is not C limited. Considering sources of C for prebiotic chemistry from hydrothermal systems, meteorites, and atmospheric deposition, this seems far easier to achieve in a shallow water body than a deep one. Such mechanisms may have located prebiotic productivity to the photic zone in such a way that the parallels to the present-day sustainability of P in deep versus shallow lakes remain highly relevant.

While it will take future kinetic models to determine how exactly prebiotic chemical networks perturb their environments, it seems almost certain that at least pre-enzymatic prebiotic chemistry will be less efficient at P uptake than life. Our analysis suggests that a Mono Lake scenario is potentially a suitable host for initiating and sustaining prebiotic chemistry that is relatively inefficient at taking up P, which may encompass a very high proportion of all prebiotic chemical networks that have so far been proposed.

**Fig. 9.**
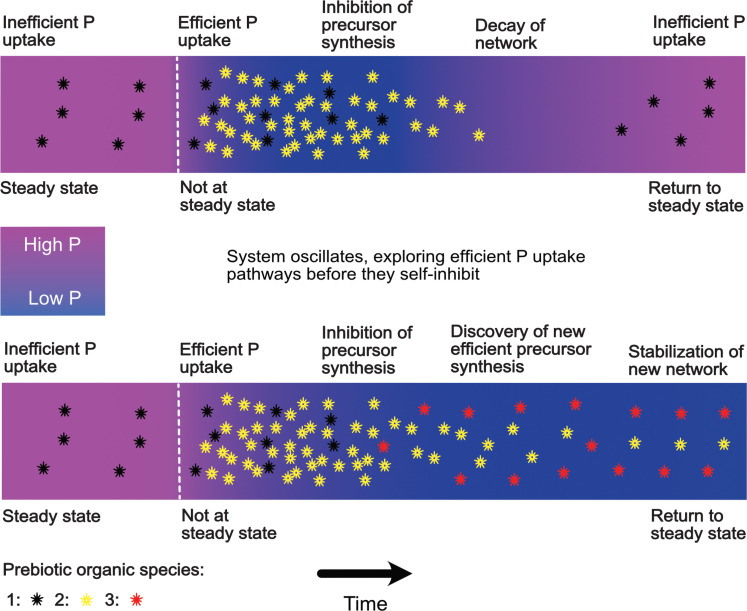
Self-inhibition of upstream and downstream chemistry in closed basins. More complex consequences of the nutrient starvation of prebiotic chemical networks may be envisaged in closed basins. Here, we compare and contrast two different outcomes of a scenario where a strongly P concentration–dependent reaction produces molecules that begin to take up P with high efficiency, i.e., in a manner not so strongly dependent on dissolved P concentration. In the first case, this leads to depletion of the basin in dissolved P and shuts down the P-dependent synthesis of their precursor molecules. The P-scavenging molecules begin to decline in number in the absence of resupply. P concentrations increase again, and we return to the initial steady-state P concentration and simple chemistry. In the second case, the system finds a way to continue producing alternative precursor molecules despite low P concentrations, e.g., enzymatic catalysis (*34*). A different steady state is reached with equal prebiotic productivity to the initial state but with lower dissolved P concentrations in the basin.

The constraints that exist within closed basin environments on the development of efficient P using prebiotic chemistry may enhance the probability of an origin of life event occurring in such an environment. We have earlier discussed simple mechanisms that can be expected to have regulated P concentrations at steady state in closed-basin lakes. In Fig. 9, we overview one way in which these mechanisms may have offered a given closed basin numerous dice rolls of prebiotic chemical network assembly and disassembly.

Consider that a strongly P concentration–dependent reaction produces molecules that begin to take up P with higher efficiency, i.e., in a manner not so strongly dependent on dissolved P concentration (Fig. 9). This would lead to depletion of the basin in dissolved P and in so doing shut down the P-dependent synthesis of the precursor molecules. The efficient P-scavenging molecules would then begin to decline in number because of the absence of resupply. P concentrations would increase, and we would see a return to the initial steady-state P concentration, at which point the simple chemistry that depends on high P concentrations would resume.

Alternatively, after an arbitrary number of cycles through the aforementioned dead end, the system may find a way to continue producing alternative precursor molecules despite low P concentrations, e.g., enzymatic catalysis and activation chemistry (*8*). A different steady state would then be reached with equal prebiotic productivity to the initial state but with lower dissolved P concentrations in the basin and a fundamentally different chemical network. In this scenario, our chemical network would have escaped its initial dependency on high steady-state P concentrations.

We can therefore see that high-inflow closed basins may naturally provide multiple bites at the prebiotic cherry: with chemical dead ends giving rise to the necessarily P-rich initial conditions of prebiotic synthesis up until a permanent way out of that condition is found. High inflow closed basins benefit not only from this resilient restocking behavior but also from a high throughput of nutrients allowing for the steady-state synthesis of a large mass of prebiotically relevant molecules as well as a lack of potentially prebiotically deadly dry periods. In the future, experiments and theoretical models may be used to explore whether a fundamental difference exists between the types of prebiotic chemical networks that can be sustained in high-inflow versus low-inflow, shallow versus deep, and permanent versus seasonal closed-basin lakes.

### Caveats and future work

We have only considered dissolved phosphate here. However, there is a growing body of literature examining the potential utility of reduced P species and reactive phosphate sources for driving prebiotic chemistry (*21*, *31*–*37*). These species are important as their higher reactivity does not necessitate such high concentrations for prebiotic chemistry to proceed. Our approach to consider the empirical evidence for phosphate supply sustainability in lakes today does not translate well to modeling the prebiotic supply rate of reduced P species or reactive phosphates, since these are not common in present-day soda lake environments according to presently published analyses (*38*). However, this may not have been the case on early Earth.

Hydrothermal, meteoritic, and volcanic sources of reduced and reactive P have all been suggested. In each case, consistent supply of any such reduced and reactive P sources seems more plausible for large inflow and potentially hydrothermal active permanent lakes than to seasonal lakes, especially those with ultra-reducing hydrothermal systems (*39*, *40*). In other words, many of the arguments made here for dissolved phosphate likely translate to reduced and reactive P species, too, provided that a source was present. Dissolved P is useful also as a general acid base catalyst and as a chemical and pH buffer in prebiotic chemistry (*5*). Therefore, the high total P supply rate in a large inflow system like Mono Lake remains relevant even if we consider reactive P species for some branches of prebiotic chemistry.

Although we have argued here that the high input rate of P to larger lakes implies a greater capacity for high steady-state prebiotic productivity alongside high P concentrations, it may be that the best lake-type scenario is a fusion of large and small lakes. Large lakes with high P inputs often have small water bodies and dense brines around their margins that may fully evaporate or freeze on a daily basis, i.e., being highly reminiscent of systems like Last Chance Lake (*41*). It may be that prebiotic chemistry in these settings benefits from the extremely high P concentrations of, e.g., Last Chance Lake, as well as the regularly P resupply offered a wider system like, e.g., Mono Lake. We intend to pursue the geochemical study of such systems in the near future.

While the exact chemical and geological pathways by which life emerged have yet to be found, we present model results that, if high rates of P supply are taken as a prerequisite condition, strongly support localized subaerial environments as plausible settings for the origin of life. However, not all closed basins are created equal. Seasonal soda lakes are favorable for wet-dry cycling scenarios of prebiotic chemistry, whereas high-inflow permanent soda lakes are more suitable for continuous prebiotic chemistry scenarios that do not require wet-dry cycles. However, we would argue that high-inflow permanent closed-basin soda lakes have a core advantage in terms of their ability to not only initiate but also sustain prebiotic chemistry.

Prebiotic chemistry does simply rely on dissolved P. Available and sustainable supplies of C, N, S, and other elements are also required to support reaction networks. Moreover, the physicochemical conditions of the environment must also be compatible with the reactions in question. At present, there is mounting evidence that soda lakes may have supplied the elements required, in the right chemical forms, and under the right conditions (moderate pressure/temperature and circumneutral pH) to promote productive prebiotic chemistry (*13*, *20*, *42*–*44*). The mechanism of concentration for P in large inflow closed basins is essentially hydrological in nature and so will also work to concentrate other nonvolatile species. Hence, many of the arguments made here about the sustainability of P supply in these systems may readily translate to other elements.

The rise of the prebiotic chemical network that gave rise to life must have perturbed its local environment in important ways, including depletion of the aqueous solution in bioessential elements. A robust local source of these elements that was not outpaced by demand would have been key in the successful transition from chemistry to life. Internal recycling can go some way to offsetting the problem but cannot progressively grow a prebiotic mass of organics without a continuous input source of feedstock ingredients. We argue here that it is high-inflow, deep, and permanent soda lakes that are most suitable for initiating and sustaining prebiotic chemistry. Mono Lake itself is an especially prebiotically relevant case study due to its formation in an endorheic basin—of high relevance to the heavily cratered and volcanically active early Earth (*45*). We conclude that prebiotic analogs of what are today Earth’s most biologically productive ecosystems may have also given rise to the first life forms.

## MATERIALS AND METHODS

We set up a steady-state model of P sources, sinks, and recycling pathways in closed-basin lakes. Our model considers only biological P sinks imposed as a free parameter and assumes P to be well-mixed throughout the basin

$$\left[P\right]=\frac{P_{\text{in}}}{\tau_{P}V}$$ (1)

where *V* (liter) is the volume of the basin, τ*_P_* is the turnover rate of P in the basin (year^*−*1^), and *P_in_* (moles per year) is the total P input to the basin. At steady state, we can solve for the concentration of dissolved P ([*P*], millimolar). The input of P to the basin can be estimated as the product of the P concentration of inflowing waters and their total annual volume. We can also reformulate the above equations to consider internal recycling of P in the basin. First, we consider a model where P recycling is treated as a free parameter. In this treatment, at steady state, we can write

$$\left[P\right]=\frac{P_{\text{in}}}{\tau_{P}f_{r}V}$$ (2)

where *f_r_* is a coefficient describing the efficiency of P recycling and where the synthesis of P-bearing organic matter is considered to be entirely P-limited and therefore to linearly compensate for enhanced P availability due to recycling.

Input values for our simulation of a Mono Lake scenario include *f_r_* = 0.92 (*27*), *V* = 3 × 10^12^ liter, annual inflow = 7.2 × 10^10^ liter, and [*P*]*_inflow_* = 1 × 10^−3^ mM, and τ*_P_* = 0.12 (*22*), and for Deer Lake include *V* = 4 × 109 liter and [*P*]*_lake_* = 1 × 10^−2^ mM (*18*). Next, we consider a scenario in which P recycling efficiency is not a free parameter but instead estimated for a given set of conditions by following the method of Farr *et al.* (*29*). We rescale their results for the surface area of Mono Lake (181.3 km^2^). This approach yields an expression for the photolytic recycling P flux (*P_r_*) as a function of [*DOP*] as follows

$$P_{r}=\text{2E}+08\;{\left[\mathrm{DOP}\right]}^{0.9886}$$ (3)

We solve Eq. 3 assuming conservatively that P liberated by photolytic recycling of organics does not act to elevate the total productivity of the system. To estimate [*DOP*] at steady state, we can write

$$P_{\mathrm{syn}}=P_{\text{in}}$$ (4)

where *P_syn_* is the organic P synthesis flux (moles per year), and

$$\left[\mathrm{DOP}\right]=\frac{P_{\mathrm{syn}}}{\tau_{\mathrm{DOP}}\;V}$$ (5)

where *P_syn_* is the synthesis flux of organic P and τ*_DOP_* is the turnover time of DOP in the system.
